# Seasonal shedding patterns of diverse henipavirus-related paramyxoviruses in Egyptian rousette bats

**DOI:** 10.1038/s41598-021-03641-w

**Published:** 2021-12-20

**Authors:** Marinda Mortlock, Marike Geldenhuys, Muriel Dietrich, Jonathan H. Epstein, Jacqueline Weyer, Janusz T. Pawęska, Wanda Markotter

**Affiliations:** 1 Centre for Viral Zoonoses, Department of Medical Virology, University of Pretoria, Pretoria, 0001 South Africa; 2 UMR Processus Infectieux en Milieu Insulaire Tropical, 97490 Sainte-Clotilde, Reunion Island France; 3 EcoHealth Alliance, New York, NY 10001 USA; 4 Centre for Emerging Zoonotic and Parasitic Diseases, National Institute for Communicable Diseases of the National Health Laboratory Services, Johannesburg, 2131 South Africa; 5 Department of Microbiology and Infectious Diseases, School of Pathology, University of Witwatersrand, Johannesburg, 2131 South Africa

**Keywords:** Viral infection, Viral epidemiology, Viral transmission

## Abstract

Bat-borne viruses in the *Henipavirus* genus have been associated with zoonotic diseases of high morbidity and mortality in Asia and Australia. In Africa, the Egyptian rousette bat species (*Rousettus aegyptiacus*) is an important viral host in which *Henipavirus*-related viral sequences have previously been identified. We expanded these findings by assessing the viral dynamics in a southern African bat population. A longitudinal study of henipavirus diversity and excretion dynamics identified 18 putative viral species circulating in a local population, three with differing seasonal dynamics, and the winter and spring periods posing a higher risk of virus spillover and transmission. The annual peaks in virus excretion are most likely driven by subadults and may be linked to the waning of maternal immunity and recolonization of the roost in early spring. These results provide insightful information into the bat-host relationship that can be extrapolated to other populations across Africa and be communicated to at-risk communities as a part of evidence-based public health education and prevention measures against pathogen spillover threats.

## Introduction

Amidst the global coronavirus disease (COVID-19) pandemic and the detection of possible bat-associated progenitors of the causative agent, our ability to predict and prevent the future emergence and transmission of zoonotic pathogens from wildlife is essential for public health security. Not all emerging agents have global pandemic potential, but even localized epidemics might have severe and devastating impacts on public health, as exemplified by recent outbreaks of Ebola virus disease in Africa and Nipah virus disease in Southeast Asia^1,2^. One of the important measures in preventing zoonotic disease spillover is identifying the maintenance populations and ecosystems where natural reservoirs of potential zoonotic agents occur. Bats have been identified as reservoirs for many zoonotic viruses and predicted to host a significantly higher proportion than other mammals, although this is still under debate with a recent study suggesting a homogenous risk among mammalian taxonomic orders^3,4^. As such, biosurveillance for bat-borne pathogens has seen a marked increase over the last few decades and has pointedly improved our knowledge of chiropteran hosts and their associated viral diversity^5–11^.

The Egyptian rousette bat (*Rousettus aegyptiacus*) is the known natural reservoir for zoonotic viruses such as Marburg and Sosuga virus^12–15^. This species has a broad geographical distribution spanning sub-Saharan Africa and parts of North Africa, the Middle East, and Southwest Asia^16–19^. In recent years, it has been associated with various viruses belonging to at least 16 viral families, including paramyxoviruses related to the *Henipavirus* genus^5,6,9,19–25^. Bat-borne henipaviruses, Hendra and Nipah viruses, are significant human and animal pathogens due to their high morbidity and mortality rates^26^. Nipah virus has also been shown to be capable of sustained human-to-human transmission chains^27^. Although these viruses have mainly been associated with *Pteropus* bat species, recent evidence suggesting an association of Nipah virus with *Rousettus leschenaultii* from India has also been reported^28^. The detection of related henipaviruses in African bat species and serological evidence of human exposure to these viruses could indicate that spillover is occurring undetected, which raises concern given the pervasive lack of public health resources in many African countries^29^.

Henipavirus dynamics in their pteropid hosts and drivers of disease emergence have been studied in more detail for Hendra and Nipah virus in *Pteropus* spp. from Australia and South Asia, respectively. Definitive seasonality and magnitude of Hendra virus excretion pulses coinciding with disease spillover and outbreaks have been reported, although viral excretion has been documented year-round^30–33^. Excretion pulses were shown to be significantly higher during the dry winter season^31^; however, variations across latitudes were observed towards Australia's northern parts, where excretion was reported in dry and wet winter regions^32^. Winter spillover variation was found to be related to winter temperatures, which affect bat behaviors and physiology, as well as human behavior^34^. For Nipah virus, viral infections in bats were not seasonal but rather were driven by waning humoral immunity in adult bat populations. Introduction of virus to a colony where seroprevalence was below a threshold of herd immunity was a likely determinant of an outbreak within bats^35^.

Investigations into African bat-borne henipaviruses have mostly been limited to the straw-colored fruit bat (*Eidolon helvum*) with a diversity of henipa- and related viruses described—one of which, *Ghanaian bat henipavirus,* has been classified into the *Henipavirus* genus^5,29,36–38^. A longitudinal assessment of henipavirus antibodies in a captive colony of *E. helvum* bats found pups to lose their maternal antibody protection between four and twelve months following birth^39^. Evidence suggests virus maintenance at a population level and horizontal virus transmission during the pregnancy and lactation stages of the reproductive period. These findings were more recently supported through molecular assessment of viral persistence and excretion in the same captive colony of *E. helvum* bats. The results suggested viral shedding throughout the year; however, a significant seasonal pattern was detected, with peaks observed in July and January^40,41^. However, limited follow-up studies have been performed to gain information on the viral dynamics, pathogenicity, and host ecology of newly described henipaviruses and related viruses in other bat species, such as *R. aegyptiacus*.

To address the role of *R. aegyptiacus* as a natural reservoir for henipavirus and related viruses on the African continent, we performed initial biosurveillance followed by longitudinal viral excretion studies in a South African bat population. Here, we report the detection of 18 henipa- and related paramyxoviruses, seasonal excretion patterns of three putative species, and the influence of age on infection status. These findings can have implications for paramyxovirus biosurveillance and risk assessments across the vast geographical distribution of this bat species.

## Materials and methods

### Study site and regulatory requirements

This study targeted a population of *R. aegyptiacus* roosting in Matlapitsi cave in Limpopo Province, South Africa (GPS: − 24.11487, 30.12151)^42^. These bats are present at this site year-round, although population size varies depending on the time of the year, i.e., higher numbers during the reproductive season (September to February) and lower numbers over the colder months (May to August). Sampling of bats was conducted longitudinally on a near-monthly basis over several years as part of a more extensive study on zoonotic virus biosurveillance. The cave is situated in the Matlapitsi valley, close to a rural community with free-roaming livestock and fruit trees planted between human dwellings. Humans have previously frequented the cave for religious practices.

Ethical clearance for the study was granted by the University of Pretoria Animal Ethics Committee, Faculty of Veterinary Science, Onderstepoort, Pretoria, South Africa (Nos. 058-14 and 054-14) and the National Health Laboratory Service (NHLS) Animal Ethics Committee, Sandringham, Johannesburg, South Africa (No. 137/12). Permits for sample collection were obtained from the Department of Economic Development, Environment and Tourism of the Limpopo Provincial Government in South Africa (Nos. CPM006806, ZA/LP/91509 and ZA/LP/100499). Permission to conduct research on animals under section 20 of the Animal Disease Act (No. 35 of 1984) was granted by the Department of Agriculture, Land Reform and Rural Development (DALRRD) of South Africa (No. 12/11/1/1/8). All methods were performed in accordance with the relevant guidelines and regulations as stipulated by the institutional ethical approval boards.

Sample collection was performed using personal protective equipment, including Tyvek coveralls, powered air-purifying respirators, fluid-resistant boots, double-layer nitrile gloves, and leather gloves, when handling bats. Single-use items were decontaminated with sodium hypochlorite (5 g/L prepared with a 1:10 dilution of domestic bleach), double-bagged into biohazard bags and transported to the BSL4 NHLS facility for autoclaving and incineration. All reusable equipment was decontaminated with a sodium hypochlorite (5 g/L) solution and sprayed down with 70% ethanol.

Weather data were obtained from the South African Weather Service at a daily resolution for the longitudinal sampling period. The nearest weather station (Tzaneen-Westfalia Estate; ~ 42 km) and rain station (Wolkberg; ~ 14 km) were selected to calculate monthly temperature and rainfall averages (Supplementary Dataset 1).

### Sample collection and sample sets

Several sample types were used (Table 1), with initial biosurveillance conducted on archival spleen and urine samples opportunistically collected over several years (Supplementary Fig. S1). These sample types were selected based on previous findings reporting successful detection of henipavirus-related viral sequences in fruit bats^5,36,37^. Collection of these tissues and urine samples from individual bats was performed as previously described whereby bats were sedated with a mixture of ketamine and xylazine (1:2; a volume of 0.05 to 0.1 mg/g body mass) and euthanized through cardiac exsanguination^9^. As a noninvasive approach, population-level urine and fecal samples were collected monthly over more than two years to assess paramyxovirus excretion over time. Population-level urine was collected as previously described, and care was taken to collect clear urine samples not containing fecal matter^9^. Briefly, urine droplets were collected using cotton-tipped swabs or by pipetting from plastic trays placed in the cave underneath roosting bats. For the collection of population-level fecal samples, fresh droppings were collected from the cave floor underneath roosting bats. Three fecal deposits were collected using cotton-tipped swabs and pooled. Rectal swabs collected from individual bats were included in the study to determine whether specific host characteristics such as age, sex, or forearm mass index (FMI) can be correlated with infection status. The swabs were collected under anesthetics during catch-and-release sampling using a mixture of ketamine and xylazine on 1:2 ratio (a volume of 0.05 to 0.1 mg/g body mass) as previously described^9^. Sterile cotton swabs (VWR Critical Swab) were dipped in 1 × phosphate-buffered saline (PBS) before being inserted into the rectum of bats and rotated three times. All biological samples were immediately transferred into a liquid nitrogen-charged dryshipper for transport to the laboratory and stored at -80 °C until further processing.

**Table 1:** Sample selection for assessing the diversity and excretion dynamics of henipa- and related viruses in Egyptian rousette bats.

Sample set	Sample type	Number	Description	Collection date
1	Tissue^a^	302	Spleen samples from individual bats^c^	2012–2018
2	Urine^a^	58	Collected from individual bats	2013–2018
3a	Population-level urine (pooled)^b^	260	Collected from sheets placed underneath roosting bats (10 per pool)	June 2017–August 2018
3b	Population-level urine (not-pooled)^b^	475	Collected from sheets placed underneath roosting bats	September 2018–September 2019
4	Population-level fecal (pooled)^b^	871	Collected from the cave floor underneath roosting bats (3 per pool)	June 2017–September 2019
5	Rectal swabs^b^	712	Collected from individual bats	November 2017–May 2019^d^

^a^Opportunistically collected.

^b^Longitudinal monthly collection.

^c^Kidney, liver, lung and intestinal tissue tested for spleen-positive individuals.

^d^Collection of rectal swabs was not performed for January, March or December 2018.

Morphological measurements of all bats were taken, including weight, forearm length, age, and sex. The age of the bats was determined based on forearm measurements, where > 89 mm was classified as adults and ≤ 89 mm as subadults^42–44^. Each bat was marked by a 3-letter-3-digit tattoo code on the propatagium of the left ventral wing of the bat. This procedure was performed while the bats were sedated and allowed for analyses of recaptured bats. Briefly, this involved cleaning of the wing surface with AIMS animal tissue prep (AIMS™), after which the code was tattooed onto the wing. Tattoo ink (AIMS Permanent black pigment; AIMS™, USA) was injected into the skin using a seven-point round liner (7RL) needle and a Bold Monk Ordinary Tattoo Machine (GetInked, South Africa). Bats recaptured during the same sampling event as when initial marking was done were released. Upon recapture during a subsequent monthly sampling event, sample collection was repeated, and the tattoo number was documented. Detailed sample information is recorded in Supplementary Dataset 2 available online. During sampling, collection of proportional numbers representing both sexes and age groups was attempted as much as possible. However, colony structure varied over time, particularly from February to April, where noticeably higher numbers of subadults were sampled.

### Sample preparation and molecular testing

Sample processing was performed in a biosafety level 3 facility (BSL3) at the Prinshof campus, University of Pretoria, South Africa. RNA was extracted from tissue and urine samples using TRIzol® reagent (Invitrogen) according to the manufacturer’s instructions. Rectal swabs and fecal material were resuspended in 800 µl and 400 µl 1 × PBS, respectively, and vortexed briefly to ensure proper resuspension. Two hundred microliters of the suspension was inactivated with the addition of an equal volume of 2 × DNA/RNA shield solution (Zymo Research), and RNA extraction was performed using the Zymogen Quick-RNA MiniPrep Plus kit (Zymo Research) according to the manufacturer’s specifications.

Reverse transcription was performed using either the SuperScript III or IV™ reverse transcriptase enzyme (Invitrogen) with 100 ng of purified random hexamers and 5 µl extracted RNA according to the manufacturer’s recommendations. Incubation was performed at 25 °C for 5 min, 60 °C for 50 min and 85 °C for 5 min. Nucleic acid amplification was performed using *Res-Mor-Hen* (RMH) primers targeting the polymerase gene in combination with an in-house optimized two-step heminested RT-PCR assay^45^. For the first round of PCR amplification, each reaction consisted of 5 µl of cDNA, 0.2 mM RMH-F1 primer, 0.3 mM RMH-R primer, 0.4 mM dNTP mix (Thermo Scientific), 2 mM MgCl_2_, 1 × DreamTaq™ buffer, 1.25 U DreamTaq™ polymerase (5 U/µl) and nuclease-free water to a final volume of 50 µl. Amplification was performed at 94 °C for 2 min; 40 cycles of 94 °C for 15 s, 48 °C for 30 s and 72 °C for 30 s; and 72 °C for 7 min. Hemi-nested reaction setup and cycling conditions were performed as described for the first round of amplification using 0.3 mM of the RMH-F2 and RMH-R primers, excluding MgCl_2_ and nuclease-water adapted accordingly.

Products were analyzed on a 1.5% agarose gel, and positive samples were subjected to Sanger sequencing on an ABI 3500xl instrument using the BigDye Terminator v3.1 Cycle Sequencing Kit (Thermo-Fisher Scientific). For all spleen-positive individuals, tissue from the kidney, liver, lung and intestine was also tested for paramyxovirus RNA.

### Statistical analyses

#### Prevalence of excretion: effect of sample type and pooling

An overall detection frequency was calculated as the number of PCR-positive samples over the number of tested samples for each type of sample: pooled swabs, individual rectal swabs, pooled urine droplets and single droplets. Because pooling can induce an upward bias of the prevalence, we also estimated a corrected overall prevalence for pooled samples using the Prevalence package and truePrevPools function in R (v.4.0.2) as previously described^46,47^.

For urine, we tested the effect of the sampling approach (pooled vs not pooled) on the percentage positivity over the period for which the two collection methods were applied (November 2017 to May 2019). We also compared the prevalence between urine and feces to assess the effect of sample type, first using pooled urine and pooled feces samples (from June 2017 to August 2018) and then single urine and pooled feces samples (from September 2018 to May 2019). Comparisons were performed using Chi-squared tests in R.

#### Temporal excretion dynamics

Data collected from the different sample types (sample sets 3, 4 and 5; Table 1) were considered separately to investigate the excretion dynamics of henipa- and related viruses. We used generalized linear models (GLMs) in R, with the sampling date (coded as different sessions) used as an explanatory variable and a binomial error and a logit link function. For individual rectal swabs, host sex, age and FMI, and their interactions were also included in the model. The forearm mass index was determined with forearm measurements, and the weight of the bats was expressed in kg/m^2^. ANOVA of the GLMs and a chi-squared test were performed to assess the significance of the variables.

### Estimating viral diversity

To estimate viral diversity, all sequences were aligned in BioEdit (v7.2.5) and trimmed to equal length^48^. The corresponding gene regions for Hendra and Nipah viruses were obtained from complete genomes available through the National Centre for Biotechnology for Information (NCBI) and included in the alignment (GenBank accession numbers NC_001906 and NC_002728, respectively). The alignment was subsequently translated to amino acid sequences, and a similarity matrix was generated. An amino acid sequence identity of 94% shared between Hendra and Nipah virus for this region was used as a conservative cut-off value to identify putative viral species. This cut-off was used to further group sequences together for a final count of putative paramyxovirus species.

### Phylogenetic analyses of detected viral sequences

Sequence alignments of detected viral sequences and representatives from the literature were generated using BioEdit (Supplementary Tables S1 and S2)^48^. The substitution model selection for the nucleotide alignment was performed using jModelTest (v2.1.10)^49^. Bayesian phylogenetic analyses were performed using the predetermined best fit model in BEAST (v2.5) with 10,000,000 iterations sampling every 1000 trees and was checked for an effective sample size (ESS) value of > 200 to ensure convergence^50^. The final phylogeny was generated using FigTree (v1.4.2) (2006–2012 Andrew Rambaut, Institute of Evolutionary Biology, University of Edinburgh). Clades representing the same putative henipavirus or related virus species were collapsed for improved visualization.

## Results

### Paramyxoviral detection rate and temporal excretion dynamics

To investigate the detection rates, diversity, and viral excretion dynamics of henipa- and related viruses associated with *R. aegyptiacus* bats, we sampled and tested a total of 2678 samples collected between 2012 and 2019. Although the assay also targeted viruses from the *Respiro-* and *Morbillivirus* genera, only viral sequences related to the *Henipavirus* genus were detected. Initial biosurveillance performed using opportunistically collected spleen and urine samples from individual bats between 2012 and 2018 (Table 1) indicated the presence of henipa- and related viruses in this bat population. Low detection frequencies of 1.66% (n = 5) and 1.72% (n = 1) were found in the two sample types, respectively. Of the five bats with viral RNA present in splenic tissue, one additionally tested positive for RNA in the renal tissue with an identical RNA sequence reported in both.

Further investigation into the preliminary findings detected viral RNA in 4.35% of pooled roost urine samples and in 2% of fecal samples across the entire sampling period (Table 2). We found no difference in the estimated detection rates when using pooled fecal samples from beneath the colony or rectal swabs from individual bats (Chi-squared test: χ^2^_1_ = 0.520, p = 0.819). Finally, comparison of viral RNA excretion in pooled feces and urine showed a significantly higher excretion in urine, both using pooled urine samples from June 2017 to August 2018 (Chi-squared test: χ^2^_1_ = 7.380, p = 0.007) and single urine droplets from September 2018 to May 2019 (χ^2^_1_ = 11.115, p < 0.001). None of the 77 recaptured bats tested positive for paramyxovirus RNA in their rectal swabs, and inferences on changes in infection status of individual bats throughout the sampling period could not be made.

**Table 2:** Overall detection rates of henipavirus and related viral RNA in different sample types across the sampling period.

Sample type	Number tested	Number positive	Positivity	Corrected positivity	Variation over time
Population-level urine (pooled)	260	16	6.2 ± 2.9%	3.4 ± 1.3%	p < 0.001 (GLM: χ^2^_14_ = 52.479)
Population-level urine (not-pooled)	475	25	5.3 ± 2.0%	n/a	p < 0.001 (GLM: χ^2^_12_ = 64.442)
Population-level fecal (pooled)	871	46	5.3 ± 1.5%	2.3 ± 0.6%	p < 0.001 (GLM: χ^2^_27_ = 81.541)
Rectal swabs	712	12	1.7 ± 0.9%	n/a	p = 0.024 (GLM: χ^2^_15_ = 27.648)

**Figure 1:**
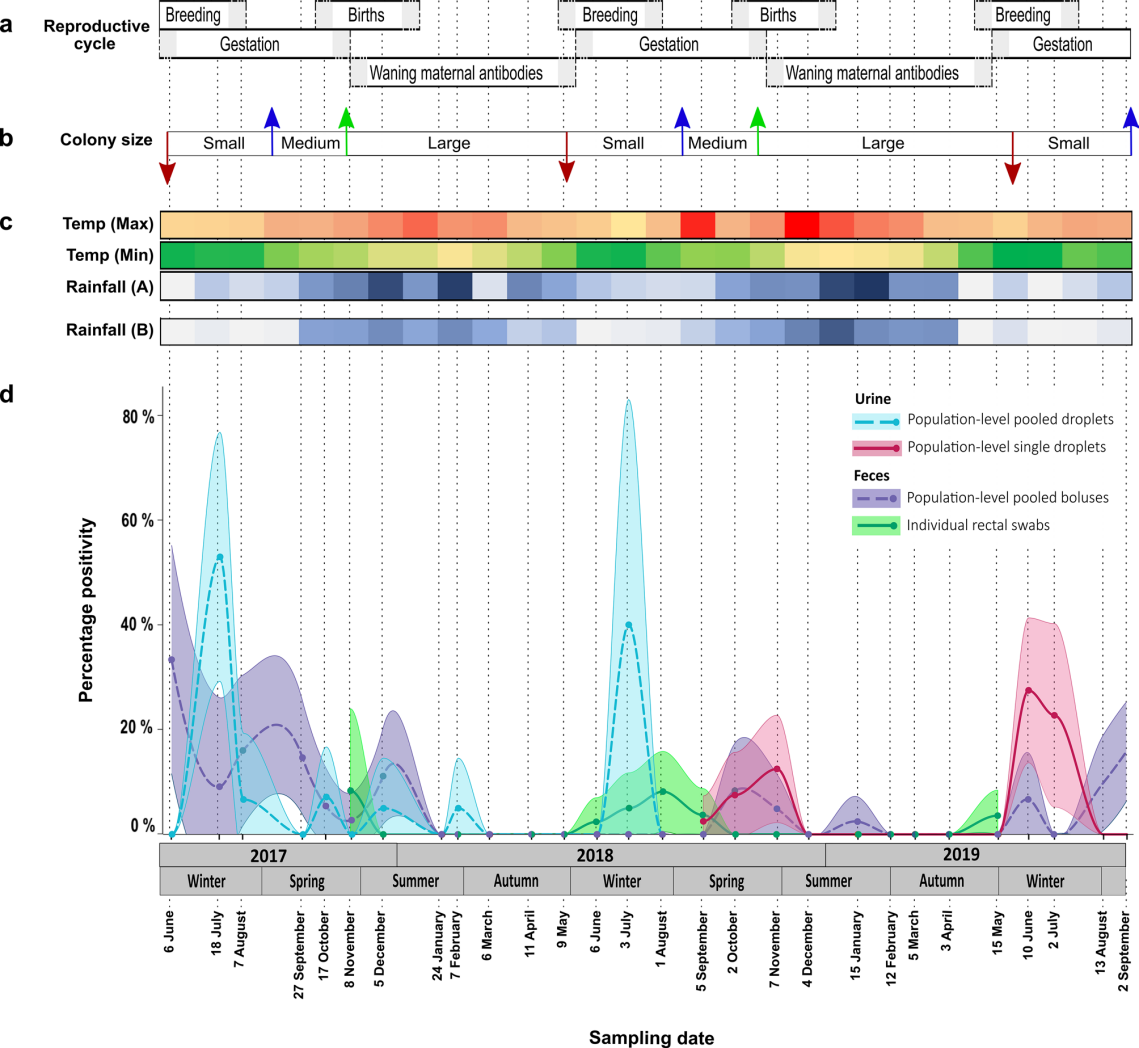
Detection of paramyxoviral RNA in a population of Egyptian rousette bats and descriptive host and environmental considerations from Matlapitsi cave, Limpopo Province, South Africa, 2017–2019. (**a**) Estimated stages over the reproductive cycle of the Egyptian rousette bat populations in South Africa (based on unpublished observations made during catch-and-release sampling and previous findings) ^42^. Shaded areas represent an early and late estimate for each stage; (**b**) Colony size variation across the sampling period defined as small—when the population size is at its lowest (~ 3000), medium—when the roost is recolonized before the birthing season (~ 7000), and large—following the birthing pulse (> 9000)^42^. Red arrows indicate the periods of mass departure from the roost near the end of May, the blue arrows represent recolonization of the roost in spring, and the green arrows indicate the influx of naïve individuals during the birthing pulse; (**c**) Monthly climate data (maximum and minimum temperatures and rainfall (A) from the closest weather station to the roost and additional rainfall (B) data from a closely situated rain station. The maximum temperature color scale bar ranges from light red to dark red (22–32.6 °C), minimum temperatures from green to light orange (8.4–19 °C), and rainfall from light to dark blue (0–168 mm); (**d**) Percentage positivity of urine, fecal and rectal swab samples for henipa- and related viral RNA detected monthly over the longitudinal sampling period calculated per monthly sampling event. Colored dots represent raw point data, with dashed lines showing values predicted by a loess function in R. Shaded areas represent 95% confidence intervals.

Assessment of the temporal excretion dynamics of the henipavirus and related viruses indicated a strong variation over time regardless of sample type (Table 2). Overall, viral RNA excretion was detected throughout most of the year, with a peak in viral excretion observed around June/July for three consecutive years (Fig. 1). Lower excretion levels were reported from September to November and seemingly decreased towards the end of each year. Notably, a cyclic absence of excretion was observed in autumn between February and May 2018 and 2019. Peak viral excretion is believed to follow in line with the waning of maternal antibodies. The viral excretion peaks also fell within the winter months for the region characterized by a cold, dry climate and limited food availability (Fig. 1). However, at a lower detection frequency, the continued excretion during the spring (September to November) coincides with the recolonization of the roost (Fig. 1).

Analyses of bat morphometric and morphological data of individuals from which rectal swabs were included indicated no effect of sex (GLM: χ^2^_1_ = 0.519, p = 0.471) or FMI (GLM: χ^2^_1_ = 1.761, p = 0.184) on infection status. A correlation between age and infection status was found, with subadults being more infected than adults (GLM: χ^2^_1_ = 6.199, p = 0.013). Although the proportion of subadults sampled was much higher in the period following the annual birth pulse (February to May), viral positives were detected during times where age classes were not skewed (Supplementary Fig. S2). This suggests that the correlation between subadults and infection status was not affected by the seasonal abundance of subadults.

### Viral diversity and phylogenetic analyses

**Figure 2:**
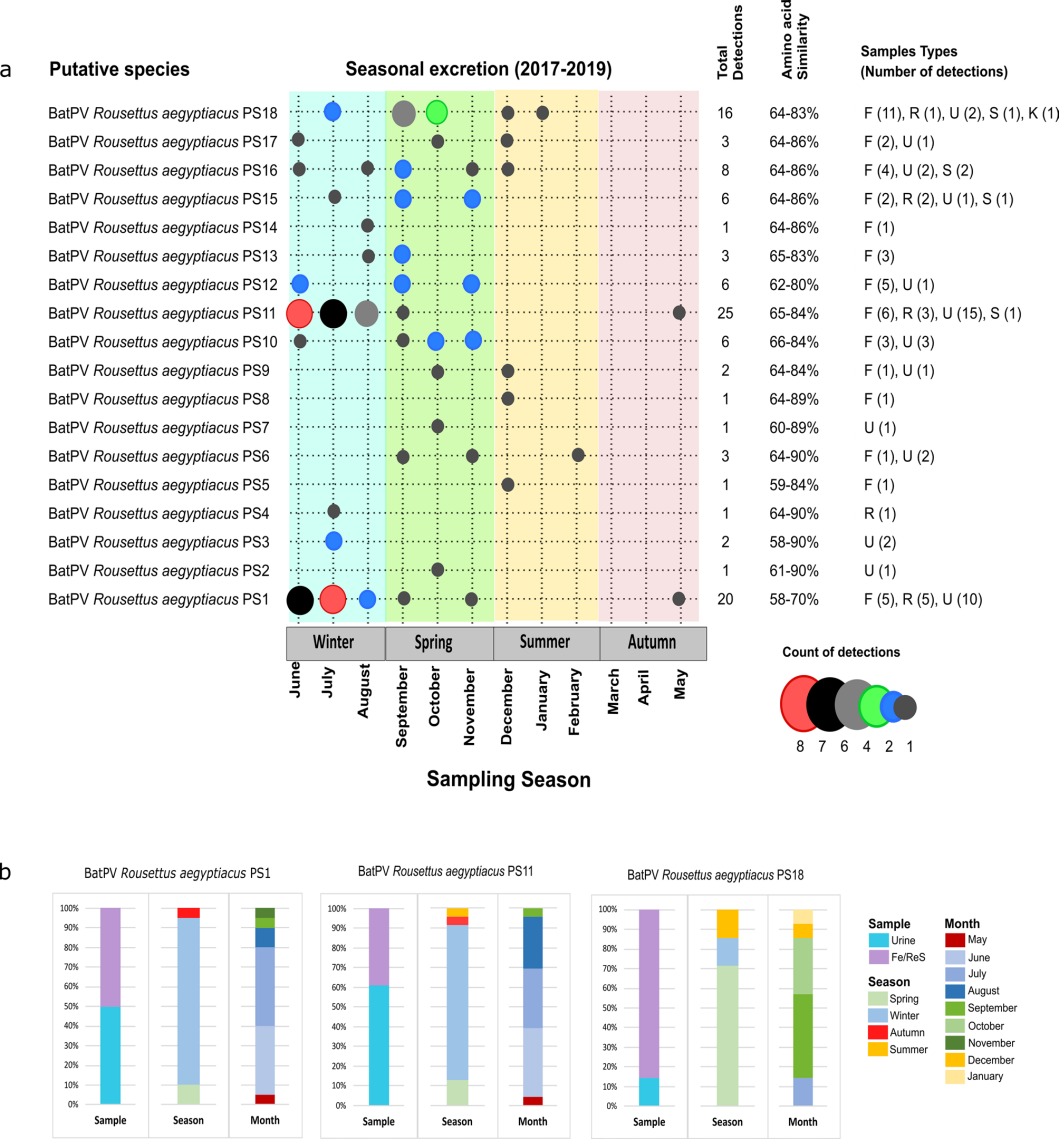
Putative henipavirus and related virus species detected in an Egyptian rousette bat population at Matlapitsi cave, Limpopo Province, South Africa. a. Seasonal excretion of putative viral species indicated in different color bars per season (sampling period June 2017 to September 2019). The number of detections is represented by dots of different sizes and colors. The total number of detections represents both the initial tissue-based biosurveillance and longitudinal excretion data. Amino acid similarities were based on a 150 amino acid sequence and indicate the similarity shared between the putative species. Sample types are F—fecal, R—rectal swabs, U—urine, S—spleen, and K—kidney. b. Bar charts represent the proportion of RNA-positive samples for putative species (PS) 1 (n = 20), PS11 (n = 23), and PS18 (n = 14) detected in bat excreta specific to sample type, season, and month of detection. For sample type, detections for fecal (Fe) and rectal swabs (ReS) were combined, as they form part of the same excretion pathway.

The henipa- and related viral diversity in *R. aegyptiacus* have not been considered extensively in previous biosurveillance studies. Assessment of the amino acid similarity between 106 viral sequences detected in this study indicated the presence of 18 putative viral species (PS) using a conservative estimate of 94% similarity at the amino acid level (Fig. 2). The overall similarity shared between putative viral species ranged from 58 to 90%, with Bat *Rousettus aegyptiacus* PS1 being most divergent at 58–70% similarity shared. Sequences highly similar (up to 99.3% amino acid similarity) to Bat *Rousettus aegyptiacus* PS1, PS11, PS12 and PS16 were previously reported in the same bat species from Kenya, Ghana and Rwanda^5,6,21^. The remaining 14 putative viral species are newly described and contribute to paramyxovirus diversity in Egyptian rousette bats. Most putative viral species were observed at a very low frequency and detected on less than 10 occasions throughout the study.

Three of the putative henipavirus and related viral species, BatPV *Rousettus aegyptiacus* PS1, PS11 and PS18, were the most frequently detected in bat excretions, being recorded 20, 23 and 14 times, respectively (Fig. 2). When considering the sample type and time of sampling, the profiles of excretion were similar for BatPV *Rousettus aegyptiacus* PS1 and PS11. These two species were detected near equally in both urine and feces/rectal swabs and predominantly in the winter months of June and July. In contrast, BatPV *Rousettus aegyptiacus* PS18 was more frequently detected in feces/rectal swabs and appeared later in the year during the spring months. These three species drive the excretion peaks observed in winter and spring, respectively (Fig. 1).

**Figure 3:**
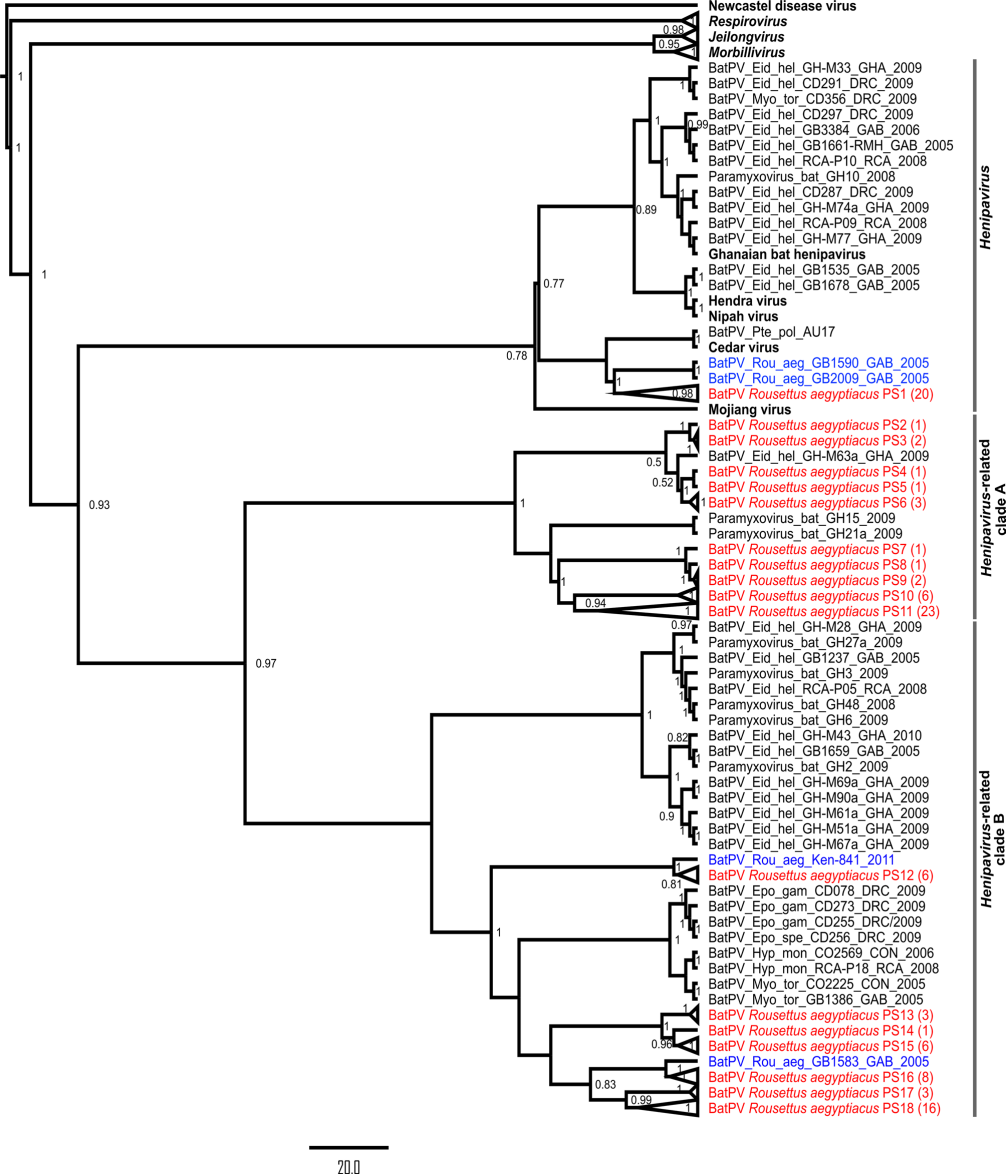
Bayesian phylogenetic analyses of partial paramyxovirus polymerase (L) gene sequences detected in Egyptian rousette bats. The proportional phylogeny was based on an alignment of sequences with 439 nucleotide lengths. Bayesian phylogenetic analysis was performed using the TIM3 + I + G substitution model with a resulting effective sample size (ESS) value of 594.3. Sequence names in red represent sequences detected in this study, blue—sequences detected in the same bat species from other countries, and black—representative sequences from the literature. Numbers in brackets at the end of red sequence names indicate the number of detections per sequence. Posterior probabilities of more than 0.5 are indicated at internal nodes, and large clades were collapsed. Newcastle disease virus was selected as an outgroup, and other *Orthoparamyxovirinae* genera are indicated in bold italics. GenBank accession numbers for all sequences used in the phylogenetic analyses are provided in Supplementary Tables S1 and S2.

All sequences detected phylogenetically grouped with the *Henipavirus* genus or in closely related sister clades, and none with the *Respiro-* or *Morbillivirus* genera which was also targeted by the assay. Analysis indicated that BatPV *Rousettus aegyptiacus* PS1, which is most diverse from the other detected sequences, groups among viruses within the *Henipavirus* genus (Fig. 3). Putative species BatPV *Rousettus aegyptiacus* PS2 to PS11 group together in the *Henipavirus-*related clade A and PS12 to PS18 in *Henipavirus-*related clade B. Viruses grouping in *Henipavirus*-related clade B were overall more prevalent in gastrointestinal excretions—as described for BatPV *Rousettus aegyptiacus* PS18. However, a definite association with this route of excretion cannot be made across the clade due to the overall low detection of the associated putative viral species (Fig. 2). As reported for the putative species analyses, some of the viral sequences grouped closely with those detected in the same host species from different African countries. The clustering of viruses specific to certain bat species/genera corresponds to previous hypotheses of host specificity of bat-borne paramyxoviruses^5,6^.

## Discussion

Biosurveillance studies in wildlife significantly contribute to the identification of viral diversity and unrecognized host species. Cross-sectional studies are often limited due to sample type bias, time of sampling and sample sizes that are not optimal for detecting and assessing viral diversity. As such, there is a need for more targeted longitudinal biosurveillance to adequately address research questions related to bat-borne viral diversity, maintenance, and dynamics within bat populations. Findings associating *R. aegyptiacus* bats with henipaviruses have been limited, with only seven detections reported from populations around Equatorial Africa^5,6,20,21^. By focusing our research on this bat species through maximizing our sample sizes and adding a temporal component, we were able to obtain high-resolution data and insight into the dynamics of henipa- and related virus diversity.

Findings from our biosurveillance support and supplement previous detections of henipa- and related viruses and expand the known virus diversity with at least 14 putative henipa- and related virus species. This is considerably higher than that reported for viruses belonging to other viral families, such as the *Filo-, Adeno-, Herpes-* and *Coronaviridae* (Geldenhuys et al. in preparation), detected in the same target population^14,51,52^. High viral diversity and strain detection rates increase the probability of strains with zoonotic potential present in these populations, as exemplified by henipaviruses and Sosuga pararubulavirus, which are observed at a higher frequency in their respective bat reservoirs^3,12,30^. In conjunction, the repeat detection of viral RNA across years could be an indication of the establishment and maintenance of these viruses within their natural reservoir populations and increased intraspecies transmissibility. The risk of human and livestock exposure or disease spillover might be considered much lower for the diversity documented at a very low detection rate; however, this diversity should not be disregarded, as their zoonotic potential remains unknown. The three dominant putative species presenting with much higher detection frequencies provide the opportunity for increased exposure and potential spillover, posing a higher risk for the local community. Moreover, the detection of these viruses in bat excreta highlights the potential for environmental contamination not only in the cave where these bats roost but also across their foraging range, as previously demonstrated for Nipah virus^53^.

Serological investigations into henipavirus dynamics in *E. helvum* bats in Africa provided initial insight into the association of these viruses with African fruit bat species^39,54^. These studies primarily relied on the cross-reactivity of African henipaviruses with Nipah virus and reported the limitation of not working with a fully characterized host–pathogen system^39^. Studies considering the specific drivers of the dynamics of unclassified bat-borne viral diversity in their associated bat species are lacking. Although some drivers are considered common for many viruses, such as the effect of season and reproduction, regional specific drivers can additionally interplay and affect virus dynamics and spillover potential. This is exemplified by the presence of Marburg virus in South African populations of *R. aegyptiacus* without local disease emergence—possibly due to the absence of extrinsic factors driving outbreaks in other parts of Africa such as hunting for and consumption of bat bushmeat or entering of caves for guano mining^14,51^. With our longitudinal molecular approach, we were able to study the dynamics of African henipa- and related viruses in a single host species. Compared to previous reports, synchronized shedding of multiple putative viral species was observed yearly, although dominant putative viral species displaying variable seasonality were detected^9,41,55^. Similar findings have been reported from a captive population of *E. helvum* bats in Ghana, whereby distinct paramyxoviral sequences display various shedding patterns^41^. This suggests the involvement of different local drivers for various paramyxovirus taxa, highlighting the limitations of lower resolution studies where such differences might be overlooked.

The temporal dynamics of BatPV *Rousettus aegyptiacus* PS1 and PS11 are in line with data previously reported for Hendra virus with a characteristic winter seasonality for peak viral excretion^31^. During this time, the bat population at Matlapitsi cave is at its lowest (Fig. 1), with most of the population exiting the roost to overwinter in other locations with a warmer climate and where food is likely more readily available^42^. For the remaining individuals, low food availability and dependance on alternate food sources, such as cultivated fruit, could result in nutritional stress. This has previously been suggested to have a physiological impact on bats and reduced immunocompetence, making them susceptible to infection^30,56^. Intraspecies transmission and exposure of local humans and livestock populations to bat-borne paramyxoviruses are more likely to occur in winter due to the aggregation of *R. aegyptiacus* bats around limited food sources, and these bats potentially feeding on cultivated fruiting trees planted throughout human settlements, as observed over the course of field studies in the area.

Although colder winter temperatures and nutritional stress may intensify pulses of viral shedding, the observation of a period of no detectable excretion before the observed peak in the winter months coincides with the influx of naïve individuals (subadults) into the colony following the birth pulse and suggests the involvement of maternal antibody protection (Fig. 1). Waning of maternal immunity provides a synchronized influx of naïve individuals who could be exposed to these viruses, resulting in large-scale horizontal transmission, infection, and virus detection during these pulses. The birthing pulse for the targeted South African population of *R. aegyptiacus* in this study was documented in late spring (October/November), which is approximately six months before the peak in viral excretion^42^. These data correspond to serological findings of the loss of henipavirus maternal antibodies in *E. helvum* bats at four to 12 months (averaged at six months) following parturition^39,54^. While our study did not include a serological aspect to confirm the influence of maternal immunity on infection acquisition during the study period, our molecular findings seem to support a greater risk of infection with henipavirus and related viruses following the loss of maternal immunity.

A difference in seasonality was described for BatPV *Rousettus aegyptiacus* PS18, with excretion predominantly detected in the spring, coinciding with the recolonization of the roost. The detection of this putative viral species predominantly upon roost colonization could represent the viral species diversity in other roosts that are reintroduced here with the influx of infected individuals into the colony^57^. However, since this is an open population with regular movement in and out of the colony to one or more different roosts, the possibility of a large metapopulation across southern Africa should be considered before such inferences can be made^42^. It becomes increasingly important to study bat movement patterns and collect spatiotemporal virological and serological data across the distribution of this bat species. Viral interspecies interactions could also account for a shift in the seasonality of excretion between the virus taxa, as previously demonstrated for rhinovirus and parainfluenza virus coinfections in humans^58^.

Our study provides some considerations on how enzootic henipavirus and related viruses in *R. aegyptiacus* circulate within a southern population and the associated exposure risk in this area. At a local scale, the period with the highest risk for human and livestock exposure to henipavirus and related viruses was determined to be winter through spring. When the natural wild fig food source of *R. aegyptiacus* is limited during winter, increased human contact is possible when bats seek other food sources, such as the cultivated fruit trees found between human dwellings within the area. In addition, *R. aegyptiacus* has been documented to expand their home ranges in winter and forage further away from the roosts, which results in increased dispersion to other settlements within the greater area^17^. The Tzaneen region northeast of the cave is known for its tropical and subtropical agriculture with cultivated fruits as one of their main lines of produce—which could serve as a secondary food source for these bats. Therefore, risk assessments for winter months should include settlements within a wider radius around the cave roost. Overwintering roosts also warrant consideration for risk assessments due to the observed increase in viral shedding during this time and the associated infection status of subadults participating in the movement between roosts. This highlights the need to incorporate bat tracking in larger-scale studies to identify secondary roosts. During spring, when the roost is recolonized and the fig trees start to fruit, the now larger population of bats will remain mostly localized and feed and within the Matlapitsi valley, increasing contact rates with humans and free-roaming livestock. Consequently, the landscape structure, bat movement and feeding behaviour, proximity of the human settlement to a roosting cave, and human behaviour might be the most important risk factors to consider when performing risk assessments and providing recommendations to the local community.

Extrapolating these conclusions to other populations of *R. aegyptiacus* bats across its geographical distribution should be made with caution, as the viral dynamics might be markedly different due to differences in life-history traits and environmental conditions. Egyptian rousette populations closer to the equator, such as those in Uganda, have bi-annual birthing pulses, which is likely due to bimodal rainfall and the year-round availability of food in these tropical regions^59,60^. As such, there will be two periods where an influx of naïve individuals into the population is observed. If the waning of maternal immunity is a driver of henipavirus and related virus dynamics within this bat species, two peaks in viral excretion would be expected within a short succession. However, if climatic conditions such as dry winter periods and nutritional stress are determining factors, then the expected excretion peaks would likely not be as pronounced. In contrast, the most southern population of *R. aegyptiacus*, documented on Table Mountain in South Africa, occurs in a region characterized by a wet-cold winter climate. This population is not believed to migrate to other roosts and represents a more closed population structure^61^. Assessing these intrinsic and extrinsic factors at a local scale will ultimately provide the data necessary to understand henipavirus and related virus dynamics in *R. aegyptiacus* bats across their distribution and aid in the movement towards more spatiotemporal predictions of disease emergence hotspots.

## Supplementary Information

Supplementary Information 1.

Dataset S1.

Dataset S2.
