# Supplementary material for: Seasonal shedding patterns of diverse henipavirus-related paramyxoviruses in Egyptian rousette bats
Source: Sci Rep. 2021 Dec 20;11:24262. doi: 10.1038/s41598-021-03641-w (PMC8688450; doi:10.1038/s41598-021-03641-w)
Supplement: Supplementary file 1 — Supplementary Information 1. [file 41598_2021_3641_MOESM1_ESM.pdf]

Seasonal shedding patterns of diverse henipavirus-related paramyxoviruses in Egyptian rousette bats

Supplemental material

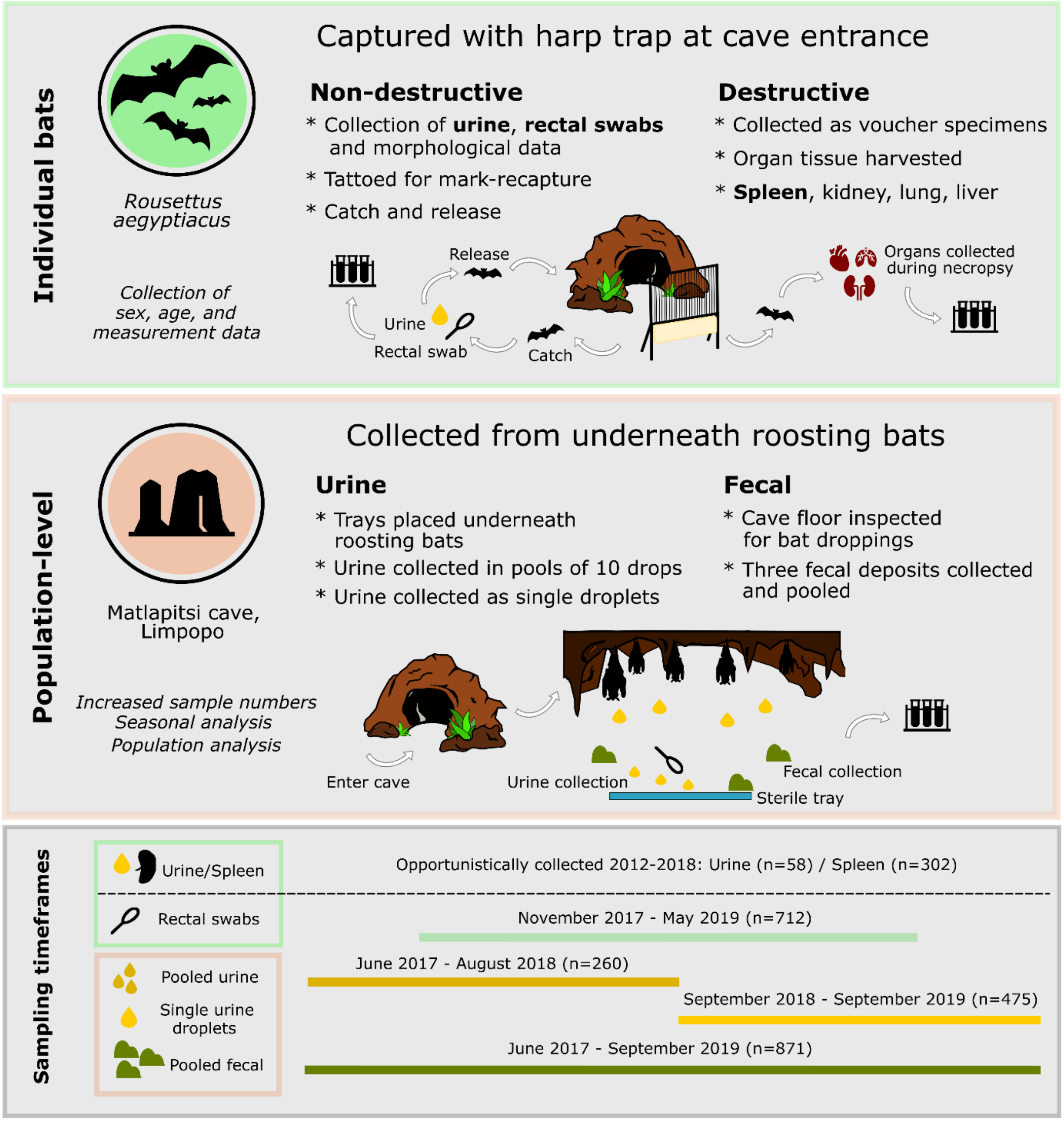

Figure S1: Infographic indicating sample collection and sample types used for paramyxovirus surveillance in *Rousettus aegyptiacus*.

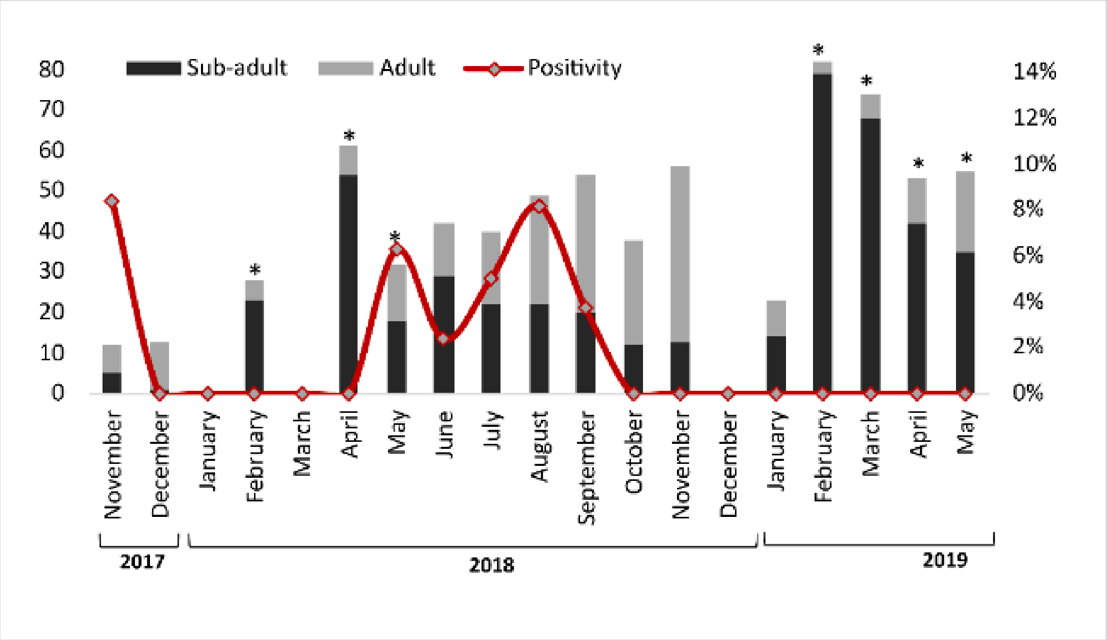

Figure S2: Graphic representation of rectal swab sample structure based on the age classes of Egyptian rousette bats. The number of bats sampled per age class across the sampling period is represented by a stacked bar. Asterisks represent months where the age ratio was skewed towards sub-adults and is reflective of the colony structure during that time of the year.

Table S1: Viral sequences used for phylogenetic analyses

Viral species (virus name)	GenBank accession number
**Outgroup**	
Avian orthoavulavirus 1 (Newcastle disease virus)	NC_002617
Respirovirus	
Bovine respirovirus 3	NC_002161
Human respirovirus 3	NC_001796
Murine respirovirus	NC_001552
Porcine respirovirus 1	NC_025402
Human respirovirus 1	NC_003461
Morbillivirus	
Measles morbillivirus	NC_001498
Rinderpest morbillivirus	NC_006296
Phocine mornillivirus	NC_028249
Canine morbillivirus	NC_001921
Dolphin morbillivirus	NC_005283
Small ruminant morbillivirus	NC_006383
Feline morbillivirus	NC_025264
Jeilongvirus	
Jun jeilongvirus	NC_007454
Beilong jeilongvirus	NC_025355
Tailam jeilongvirus	NC_007803
Henipavirus	
Cedar henipavirus (Cedar virus)	NC_025351
Ghanaian bat henipavirus (Ghana virus)	NC_025256
Hendra henipavirus (Hendra virus)	NC_001906
Mojiang henipavirus (Mòjiāng virus)	NC_025352
Nipah henipavirus (Nipah virus)	NC_002728
**Unclassified from literature**	
BatPV_Pte_pol_AU17	KF871302
BatPV_Eid_hel_GB1535_GAB_2005	HQ660141
BatPV_Eid_hel_GB1678_GAB_2005	HQ660144
Paramyxovirus_bat_GH21a_2009	FJ971939
BatPV_Rou_aeg_Ken-893_2011	KC578633
BatPV_Eid_hel_GH-M63a_GHA_2009	HQ660136
BatPV_Rou_aeg_Ken-841_2011	KC578634
BatPV_Myo_tor_CO2225_CON_2005	HQ660118
BatPV_Myo_tor_GB1386_GAB_2005	HQ660137
BatPV_Hyp_mon_CO2569_CON_2006	HQ660094
BatPV_Hyp_mon_RCA-P18_RCA_2008	HQ660152
BatPV_Epo_gam_CD255_DRC/2009	HQ660120
BatPV_Epo_spe_CD256_DRC_2009	HQ660121
BatPV_Epo_gam_CD273_DRC_2009	HQ660122
BatPV_Epo_gam_CD078_DRC_2009	HQ660128
BatPV_Rou_aeg_GB1583_GAB_2005	HQ660138
Paramyxovirus_bat_GH15_2009	FJ971935
Paramyxovirus_bat_GH48_2008	FJ609194
Paramyxovirus_bat_GH6_2009	FJ971945
BatPV_Eid_hel_RCA-P05_RCA_2008	HQ660150
Paramyxovirus_bat_GH3_2009	FJ609192
BatPV_Eid_hel_GB1237_GAB_2005	HQ660140
Paramyxovirus_bat_GH27a_2009	FJ971940
BatPV_Eid_hel_GH-M28_GHA_2009	HQ660147
Paramyxovirus_bat_GH2_2009	FJ971944
BatPV_Eid_hel_GB1659_GAB_2005	HQ660142
BatPV_Eid_hel_GH-M67a_GHA_2009	HQ660131
BatPV_Eid_hel_GH-M51a_GHA_2009	HQ660132
BatPV_Eid_hel_GH-M61a_GHA_2009	HQ660133
BatPV_Eid_hel_GH-M90a_GHA_2009	HQ660134
BatPV_Eid_hel_GH-M69a_GHA_2009	HQ660135
BatPV_Eid_hel_GH-M43_GHA_2010	HQ660127
BatPV_Eid_hel_GH-M74a_GHA_2009	HQ660129
BatPV_Eid_hel_GH-M77_GHA_2009	HQ660130
BatPV_Eid_hel_RCA-P09_RCA_2008	HQ660151
BatPV_Eid_hel_CD287_DRC_2009	HQ660123
Paramyxovirus_bat_GH10_2008	FJ609191

BatPV_Eid_hel_CD297_DRC_2009	HQ660125
BatPV_Eid_hel_GB1661-RMH_GAB_2005	HQ660143
BatPV_Eid_hel_RCA-P10_RCA_2008	HQ660149
BatPV_Eid_hel_GB3384_GAB_2006	HQ660146
BatPV_Eid_hel_CD291_DRC_2009	HQ660124
BatPV_Myo_tor_CD356_DRC_2009	HQ660126
BatPV_Eid_hel_GH-M33_GHA_2009	HQ660148
BatPV_Rou_aeg_GB1590_GAB_2005	HQ660139
BatPV_Rou_aeg_GB2009_GAB_2005	HQ660145

Table S2: Information on putative viruses detected in this study

Sequence	Putative species	Range with other	Sample type	Year	Month	Season	Wet/Dry	Accession number
BatPV_R_aeg_RSA-10028ReS_2018	BatPV Rousettus aegyptiacus PS18	64-83%	Rectal swab	2018	September	Spring	Dry	MZ275509
BatPV_R_aeg_RSA-11409ReS_2019	BatPV Rousettus aegyptiacus PS11	65-83%	Rectal swab	2019	May	Autumn	Dry	MZ275510
BatPV_R_aeg_RSA-11414ReS_2019	BatPV Rousettus aegyptiacus PS1	58-70%	Rectal swab	2019	May	Autumn	Dry	MZ275511
BatPV_R_aeg_RSA-8388ReS_2017	BatPV Rousettus aegyptiacus PS15	64-86%	Rectal swab	2017	November	Spring	Wet	MZ275499
BatPV_R_aeg_RSA-9688ReS_2017	BatPV Rousettus aegyptiacus PS1	58-70%	Rectal swab	2018	June	Winter	Dry	MZ275500
BatPV_R_aeg_RSA-9716ReS_2018	BatPV Rousettus aegyptiacus PS1	58-70%	Rectal swab	2018	July	Winter	Dry	MZ275501
BatPV_R_aeg_RSA-9717ReS_2018	BatPV Rousettus aegyptiacus PS1	58-70%	Rectal swab	2018	July	Winter	Dry	MZ275502
BatPV_R_aeg_RSA-9759ReS_2018	BatPV Rousettus aegyptiacus PS15	64-86%	Rectal swab	2018	July	Winter	Dry	MZ275503
BatPV_R_aeg_RSA-9760ReS_2018	BatPV Rousettus aegyptiacus PS4	64-90%	Rectal swab	2018	July	Winter	Dry	MZ275504
BatPV_R_aeg_RSA-9819ReS_2018	BatPV Rousettus aegyptiacus PS11	65-84%	Rectal swab	2018	August	Winter	Dry	MZ275507
BatPV_R_aeg_RSA-9824ReS_2018	BatPV Rousettus aegyptiacus PS1	58-70%	Rectal swab	2018	August	Winter	Dry	MZ275508
BatPV_R_aeg_RSA-9996ReS_2018	BatPV Rousettus aegyptiacus PS11	65-84%	Rectal swab	2018	September	Spring	Dry	MZ275465
BatPV_R_aeg_RSA-UPE064Fe_2017	BatPV Rousettus aegyptiacus PS11	65-84%	Faecal	2017	June	Winter	Dry	MZ275461
BatPV_R_aeg_RSA-UPE068Fe_2017	BatPV Rousettus aegyptiacus PS16	64-86%	Faecal	2017	June	Winter	Dry	MZ275462
BatPV_R_aeg_RSA-UPE070Fe_2017	BatPV Rousettus aegyptiacus PS17	64-86%	Faecal	2017	June	Winter	Dry	MZ275473
BatPV_R_aeg_RSA-UPE075Fe_2017	BatPV Rousettus aegyptiacus PS12	62-80%	Faecal	2017	June	Winter	Dry	MZ275474
BatPV_R_aeg_RSA-UPE076Fe_2017	BatPV Rousettus aegyptiacus PS10	66-84%	Faecal	2017	June	Winter	Dry	MZ275475
BatPV_R_aeg_RSA-UPE077Fe_2017	BatPV Rousettus aegyptiacus PS12	62-80%	Faecal	2017	June	Winter	Dry	MZ275476
BatPV_R_aeg_RSA-UPE1018Fe_2018	BatPV Rousettus aegyptiacus PS18	64-83%	Faecal	2018	October	Spring	Wet	MZ275496
BatPV_R_aeg_RSA-UPE1019Fe_2018	BatPV Rousettus aegyptiacus PS17	64-86%	Faecal	2018	October	Spring	Wet	MZ275497
BatPV_R_aeg_RSA-UPE103Fe_2017	BatPV Rousettus aegyptiacus PS18	64-83%	Faecal	2017	July	Winter	Dry	MZ275477
BatPV_R_aeg_RSA-UPE1041Ur_2018	BatPV Rousettus aegyptiacus PS2	61-90%	Urine	2018	October	Spring	Wet	MZ275513
BatPV_R_aeg_RSA-UPE1047Ur_2018	BatPV Rousettus aegyptiacus PS9	64-84%	Urine	2018	October	Spring	Wet	MZ275514
BatPV_R_aeg_RSA-UPE1050Ur_2018	BatPV Rousettus aegyptiacus PS10	66-84%	Urine	2018	October	Spring	Wet	MZ275515
BatPV_R_aeg_RSA-UPE1100Fe_2018	BatPV Rousettus aegyptiacus PS12	62-80%	Faecal	2018	November	Spring	Wet	MZ275498
BatPV_R_aeg_RSA-UPE112Ur(a)_2017	BatPV Rousettus aegyptiacus PS11	65-84%	Urine	2017	July	Winter	Dry	MH259199
BatPV_R_aeg_RSA-UPE1131Fe_2018	BatPV Rousettus aegyptiacus PS16	64-84%	Faecal	2018	November	Spring	Wet	MZ275463
BatPV_R_aeg_RSA-UPE1136Ur_2018	BatPV Rousettus aegyptiacus PS10	66-84%	Urine	2018	November	Spring	Wet	MZ275516
BatPV_R_aeg_RSA-UPE113Ur(a)_2017	BatPV Rousettus aegyptiacus PS1	58-70%	Urine	2017	July	Winter	Dry	MH259200
BatPV_R_aeg_RSA-UPE1143Ur_2018	BatPV Rousettus aegyptiacus PS10	66-84%	Urine	2018	November	Spring	Wet	MZ275517
BatPV_R_aeg_RSA-UPE1147Ur_2018	BatPV Rousettus aegyptiacus PS6	64-90%	Urine	2018	November	Spring	Wet	MZ275518
BatPV_R_aeg_RSA-UPE114Ur_2017	BatPV Rousettus aegyptiacus PS1	58-70%	Urine	2017	July	Winter	Dry	MH259201
BatPV_R_aeg_RSA-UPE1162Ur_2018	BatPV Rousettus aegyptiacus PS12	62-80%	Urine	2018	November	Spring	Wet	MZ275519
BatPV_R_aeg_RSA-UPE1163Ur_2018	BatPV Rousettus aegyptiacus PS15	64-86%	Urine	2018	November	Spring	Wet	MZ275520
BatPV_R_aeg_RSA-UPE116Ur_2017	BatPV Rousettus aegyptiacus PS11	65-84%	Urine	2017	July	Winter	Dry	MH259202
BatPV_R_aeg_RSA-UPE117a_2017	BatPV Rousettus aegyptiacus PS11	65-84%	Urine	2017	July	Winter	Dry	MH259203
BatPV_R_aeg_RSA-UPE118Ur(a)_2017	BatPV Rousettus aegyptiacus PS18	64-83%	Urine	2017	July	Winter	Dry	MH259204

BatPV_R_aeg_RSA-UPE119Ur(a)_2017	BatPV Rousettus aegyptiacus PS11	65-84%	Urine	2017	July	Winter	Dry	MH259205
BatPV_R_aeg_RSA-UPE122Ur(a)_2017	BatPV Rousettus aegyptiacus PS11	65-84%	Urine	2017	July	Winter	Dry	MH259206
BatPV_R_aeg_RSA-UPE125Ur_2017	BatPV Rousettus aegyptiacus PS11	65-84%	Urine	2017	July	Winter	Dry	MH259207
BatPV_R_aeg_RSA-UPE140Fe_2017	BatPV Rousettus aegyptiacus PS11	65-84%	Faecal	2017	August	Winter	Dry	MZ275478
BatPV_R_aeg_RSA-UPE145Fe_2017	BatPV Rousettus aegyptiacus PS11	65-84%	Faecal	2017	August	Winter	Dry	MZ275479
BatPV_R_aeg_RSA-UPE1483Fe_2019	BatPV Rousettus aegyptiacus PS18	64-83%	Faecal	2019	January	Summer	Wet	MZ275466
BatPV_R_aeg_RSA-UPE155Fe_2017	BatPV Rousettus aegyptiacus PS1	58-70%	Faecal	2017	August	Winter	Dry	MZ275480
BatPV_R_aeg_RSA-UPE157Fe_2017	BatPV Rousettus aegyptiacus PS11	65-84%	Faecal	2017	August	Winter	Dry	MZ275481
BatPV_R_aeg_RSA-UPE170Ur_2017	BatPV Rousettus aegyptiacus PS16	64-86%	Urine	2017	August	Winter	Dry	MH259208
BatPV_R_aeg_RSA-UPE1876Fe_2019	BatPV Rousettus aegyptiacus PS1	58-70%	Faecal	2019	June	Winter	Dry	MZ275521
BatPV_R_aeg_RSA-UPE1881Fe_2019	BatPV Rousettus aegyptiacus PS1	58-70%	Faecal	2019	June	Winter	Dry	MZ275522
BatPV_R_aeg_RSA-UPE1899Ur_2019	BatPV Rousettus aegyptiacus PS11	65-83%	Urine	2019	June	Winter	Dry	MZ275523
BatPV_R_aeg_RSA-UPE1900Ur_2019	BatPV Rousettus aegyptiacus PS1	58-70%	Urine	2019	June	Winter	Dry	MZ275467
BatPV_R_aeg_RSA-UPE1904Ur_2019	BatPV Rousettus aegyptiacus PS11	65-83%	Urine	2019	June	Winter	Dry	MZ275469
BatPV_R_aeg_RSA-UPE1907Ur_2019	BatPV Rousettus aegyptiacus PS11	65-83%	Urine	2019	June	Winter	Dry	MZ275470
BatPV_R_aeg_RSA-UPE1912Ur_2019	BatPV Rousettus aegyptiacus PS11	65-84%	Urine	2019	June	Winter	Dry	MZ275471
BatPV_R_aeg_RSA-UPE1913Ur_2019	BatPV Rousettus aegyptiacus PS1	58-70%	Urine	2019	June	Winter	Dry	MZ275468
BatPV_R_aeg_RSA-UPE1915Ur_2019	BatPV Rousettus aegyptiacus PS11	65-84%	Urine	2019	June	Winter	Dry	MZ275472
BatPV_R_aeg_RSA-UPE1916Ur_2019	BatPV Rousettus aegyptiacus PS1	58-70%	Urine	2019	June	Winter	Dry	MZ275524
BatPV_R_aeg_RSA-UPE1919Ur_2019	BatPV Rousettus aegyptiacus PS11	65-83%	Urine	2019	June	Winter	Dry	MZ275525
BatPV_R_aeg_RSA-UPE1927Ur_2019	BatPV Rousettus aegyptiacus PS11	65-84%	Urine	2019	June	Winter	Dry	MZ275526
BatPV_R_aeg_RSA-UPE1928Ur_2019	BatPV Rousettus aegyptiacus PS1	58-70%	Urine	2019	June	Winter	Dry	MZ275527
BatPV_R_aeg_RSA-UPE1954Ur_2019	BatPV Rousettus aegyptiacus PS3	58-90%	Urine	2019	July	Winter	Dry	MZ275505
BatPV_R_aeg_RSA-UPE1959Ur_2019	BatPV Rousettus aegyptiacus PS11	65-83%	Urine	2019	July	Winter	Dry	MZ275528
BatPV_R_aeg_RSA-UPE1963Ur_2019	BatPV Rousettus aegyptiacus PS3	58-90%	Urine	2019	July	Winter	Dry	MZ275506
BatPV_R_aeg_RSA-UPE1967Ur_2019	BatPV Rousettus aegyptiacus PS1	58-70%	Urine	2019	July	Winter	Dry	MZ275529
BatPV_R_aeg_RSA-UPE1970Ur_2019	BatPV Rousettus aegyptiacus PS1	58-70%	Urine	2019	July	Winter	Dry	MZ275530
BatPV_R_aeg_RSA-UPE2077Fe_2019	BatPV Rousettus aegyptiacus PS13	65-83%	Faecal	2019	August	Winter	Dry	MZ275531
BatPV_R_aeg_RSA-UPE207Fe_2017	BatPV Rousettus aegyptiacus PS12	62-80%	Faecal	2017	September	Spring	Dry	MZ275482
BatPV_R_aeg_RSA-UPE2099Fe_2019	BatPV Rousettus aegyptiacus PS14	64-86%	Faecal	2019	August	Winter	Dry	MZ275532
BatPV_R_aeg_RSA-UPE2111Fe_2019	BatPV Rousettus aegyptiacus PS11	65-83%	Faecal	2019	August	Winter	Dry	MZ275533
BatPV_R_aeg_RSA-UPE2115Fe_2019	BatPV Rousettus aegyptiacus PS11	65-83%	Faecal	2019	August	Winter	Dry	MZ275534
BatPV_R_aeg_RSA-UPE2117Fe_2019	BatPV Rousettus aegyptiacus PS18	64-83%	Faecal	2019	September	Spring	Dry	MZ275535
BatPV_R_aeg_RSA-UPE2128Fe_2019	BatPV Rousettus aegyptiacus PS18	64-83%	Faecal	2019	September	Spring	Dry	MZ275536
BatPV_R_aeg_RSA-UPE2130Fe_2019	BatPV Rousettus aegyptiacus PS18	64-83%	Faecal	2019	September	Spring	Dry	MZ275537
BatPV_R_aeg_RSA-UPE2133Fe_2019	BatPV Rousettus aegyptiacus PS18	64-83%	Faecal	2019	September	Spring	Dry	MZ275538
BatPV_R_aeg_RSA-UPE2144Fe_2019	BatPV Rousettus aegyptiacus PS13	65-83%	Faecal	2019	September	Spring	Dry	MZ275539
BatPV_R_aeg_RSA-UPE2148Fe_2019	BatPV Rousettus aegyptiacus PS13	65-83%	Faecal	2019	September	Spring	Dry	MZ275540
BatPV_R_aeg_RSA-UPE2160Fe_2019	BatPV Rousettus aegyptiacus PS12	62-80%	Faecal	2019	September	Spring	Dry	MZ275541
BatPV_R_aeg_RSA-UPE2166Fe_2019	BatPV Rousettus aegyptiacus PS6	64-90%	Faecal	2019	September	Spring	Dry	MZ275542
BatPV_R_aeg_RSA-UPE2167Fe_2019	BatPV Rousettus aegyptiacus PS18	64-83%	Faecal	2019	September	Spring	Dry	MZ275543

BatPV_R_aeg_RSA-UPE218Fe_2017	BatPV Rousettus aegyptiacus PS15	64-86%	Faecal	2017	September	Spring	Dry	MZ275483
BatPV_R_aeg_RSA-UPE220Fe_2017	BatPV Rousettus aegyptiacus PS16	64-86%	Faecal	2017	September	Spring	Dry	MZ275464
BatPV_R_aeg_RSA-UPE224Fe_2017	BatPV Rousettus aegyptiacus PS15	64-86%	Faecal	2017	September	Spring	Dry	MZ275484
BatPV_R_aeg_RSA-UPE230Fe_2017	BatPV Rousettus aegyptiacus PS1	58-70%	Faecal	2017	September	Spring	Dry	MZ275485
BatPV_R_aeg_RSA-UPE246Fe_2017	BatPV Rousettus aegyptiacus PS10	66-84%	Faecal	2017	September	Spring	Dry	MZ275486
BatPV_R_aeg_RSA-UPE281Fe_2017	BatPV Rousettus aegyptiacus PS10	66-84%	Faecal	2017	October	Spring	Wet	MZ275487
BatPV_R_aeg_RSA-UPE306Fe_2017	BatPV Rousettus aegyptiacus PS18	64-83%	Faecal	2017	October	Spring	Wet	MZ275488
BatPV_R_aeg_RSA-UPE323Ur_2017	BatPV Rousettus aegyptiacus PS18	64-83%	Urine	2017	October	Spring	Wet	MH259209
BatPV_R_aeg_RSA-UPE331Ur(a)_2017	BatPV Rousettus aegyptiacus PS7	60-89%	Urine	2017	October	Spring	Wet	MH259210
BatPV_R_aeg_RSA-UPE395Fe_2017	BatPV Rousettus aegyptiacus PS1	58-70%	Faecal	2017	November	Spring	Wet	MZ275489
BatPV_R_aeg_RSA-UPE426Fe_2017	BatPV Rousettus aegyptiacus PS5	59-84%	Faecal	2017	December	Summer	Wet	MZ275490
BatPV_R_aeg_RSA-UPE437Fe_2017	BatPV Rousettus aegyptiacus PS18	64-83%	Faecal	2017	December	Summer	Wet	MZ275491
BatPV_R_aeg_RSA-UPE443Fe_2017	BatPV Rousettus aegyptiacus PS9	64-84%	Faecal	2017	December	Summer	Wet	MZ275492
BatPV_R_aeg_RSA-UPE447Fe_2017	BatPV Rousettus aegyptiacus PS16	64-86%	Faecal	2017	December	Summer	Wet	MZ275493
BatPV_R_aeg_RSA-UPE463Ur_2017	BatPV Rousettus aegyptiacus PS17	64-85%	Urine	2017	December	Summer	Wet	MH259211
BatPV_R_aeg_RSA-UPE493Fe_2017	BatPV Rousettus aegyptiacus PS8	64-89%	Faecal	2017	December	Summer	Wet	MZ275494
BatPV_R_aeg_RSA-UPE612Ur_2018	BatPV Rousettus aegyptiacus PS6	64-90%	Urine	2018	February	Summer	Wet	MH259212
BatPV_R_aeg_RSA-UPE816Ur_2018	BatPV Rousettus aegyptiacus PS1	58-70%	Urine	2018	July	Winter	Dry	MH719236
BatPV_R_aeg_RSA-UPE826Ur_2018	BatPV Rousettus aegyptiacus PS1	58-70%	Urine	2018	July	Winter	Dry	MH719237
BatPV_R_aeg_RSA-UPE970Ur_2018	BatPV Rousettus aegyptiacus PS16	64-86%	Urine	2018	September	Spring	Dry	MZ275512
BatPV_R_aeg_RSA-UPE999Fe_2018	BatPV Rousettus aegyptiacus PS18	64-83%	Faecal	2018	October	Spring	Wet	MZ275495
BatPV_R_aeg_RSA-5932Sp_2015	BatPV Rousettus aegyptiacus PS1	65-84%	Spleen	2015	October	Spring	Wet	MH259195
BatPV_R_aeg_RSA-6907Ur_2016	BatPV Rousettus aegyptiacus PS1	65-84%	Urine - Individual	2016	June	Winter	Dry	MH259198
BatPV_R_aeg_RSA-5119Sp(a)_2015	BatPV Rousettus aegyptiacus PS2	64-86%	Spleen	2015	March	Autumn	Wet	MH259193
BatPV_R_aeg_RSA-5865Sp_2015	BatPV Rousettus aegyptiacus PS2	64-86%	Spleen	2015	April	Autumn	Wet	MH259194
BatPV_R_aeg_RSA-2722Sp_2013	BatPV Rousettus aegyptiacus PS3	64-83%	Spleen	2013	July	Winter	Dry	MH259191
BatPV_R_aeg_RSA-3584Sp(a)_2013	BatPV Rousettus aegyptiacus PS8	64-86%	Spleen	2013	November	Spring	Wet	MH259192
BatPV_R_aeg_RSA-2722Ki_2013	BatPV Rousettus aegyptiacus PS3	64-83%	Spleen	2013	July	Winter	Dry	MH259196
